# Shifting paradigms: how the fight for 'universal access to AIDS treatment and prevention' supports achieving 'comprehensive primary health care for all'

**DOI:** 10.1186/1744-8603-4-11

**Published:** 2008-11-18

**Authors:** Gorik Ooms

**Affiliations:** 1 Institute of Tropical Medicine, Antwerp, Belgium

## Abstract

In a recent issue of Globalization and Health, Yu et al. examine the impact of HIV/AIDS programs on health care systems. This editorial considers their position and confirms that the former actually supports the latter aim; the two approaches are not at odds with one another, but could be viewed as complementary. A key requirement towards meeting both objectives is to ensure sustained international aid.

## Commentary

During the past two years, we have witnessed the polarization between natural born allies: the proponents of universal access to AIDS treatment and prevention and the proponents of primary health care for all. With their paper '..Investment in HIV/AIDS programs: Does it help strengthen health systems in developing countries?', Yu and colleagues try to put this division to rest [1].

Their paper reads like a King Solomon verdict. Yes, the fight for universal access to AIDS treatment and prevention has caused some unintended negative side-effects for the fight for comprehensive primary health care for all. However, the evidence is mainly anecdotal, and positive synergies seem to outweigh the negative side-effects.

Both camps might react negatively to this assessment. The proponents of universal access to AIDS treatment and prevention might assert: 'There is no solid evidence whatsoever of negative side-effects!' Likewise, the proponents of primary health care for all might argue that the authors, coming from a United Nations agency tasked to deal with AIDS, underestimate the negative side-effects, and have no scientific scale that allows comparing one against the other. Both groups would be missing the main point: the fight for universal access to AIDS treatment and prevention created a new momentum for the fight for comprehensive primary health care for all.

Yu et al. assert that *"The most spectacular result of [the World Health Organization]'s "3 by 5" initiative was to demonstrate that delivering [Anti-Retroviral Treatment] through a public health approach is feasible even where health systems are weak overall." *However, they do not explicitly ask the question how this 'spectacular result' was realized. They do provide the answer when they point out that *"the majority of developing countries cannot fund [Primary Health Care] with domestic resources alone. ... sustained commitment is especially important for a disease like HIV/AIDS, where patient survival depends on lifelong access to drugs, but is also important for funding broader issues such as health systems strengthening."*

Only five years ago, such as statement would have been considered as heresy, especially if coming from the World Health Organization. Health development orthodoxy held that international health aid is temporary, aiming at 'developing' recipient countries' own 'hidden capacity'. The possibility that some countries simply do not have sufficient capacity waiting to be 'developed', because they are too poor to finance primary health care with domestic resources alone, was usually not considered.

The fight for universal access to AIDS treatment and prevention changed that paradigm. AIDS treatment was and still remains so obviously 'unaffordable' for the economies of low-income countries, that a paradigm shift imposed itself. The only way to make '3 by 5' – 3 million people receiving AIDS treatment by 2005 – sustainable was to separate *financial *sustainability from *operational *sustainability. While countries were expected to develop their capacity to manage AIDS treatment programs without external assistance, they were not expected to demonstrate their capacity to finance AIDS treatment programs. The financial sustainability of these programs relies on sustained international health aid.

This paradigm shift was the result of the realization that even at a cost of US$100 per person per year for medicines only, AIDS treatment remains unaffordable for countries with a government health expenditure budget of US$10 per person per year, and the realization that once started, AIDS treatment would have to be continued and thus requires a long-term commitment. There is a growing realization that the same is probably true for 'health systems strengthening': to hire a physician and two nurses per thousand people on a US$10 per person per year budget is quite a challenge, and the long-term commitment is required for increasing the health workforce as much as for AIDS treatment. It takes at least three years to increase the health workforce training capacity in some low-income countries; then three to six years are needed to train more nurses and physicians; and, then five to ten years contracts are needed to hire those people. To start investing in increased health workforce training capacity today, low-income countries need international health aid commitments that are valid for 15 to 20 years. Impossible? Certainly, it is not longer than the commitments required for AIDS treatment.

Ultimately, this paradigm shift will be the best service rendered by the fight for universal access to AIDS treatment and prevention, to the fight for comprehensive primary health care for all. Comprehensive primary health care for all was considered 'unaffordable' and 'unsustainable' within the old paradigm. Within the new paradigm it is not. The global economy is wealthy enough to finance comprehensive primary health care for all, of which universal access to AIDS treatment and prevention is an essential part.

If the paper of Yu and colleagues signaled a paradigm shift within the World Health Organization, it appears to be a short-lived one. The World Health Report 2008, published a few weeks after Yu's paper, acknowledges that "the steep increase in external funds directed towards health through bilateral channels or through the new generation of global financing instruments has boosted the vitality of the health sector", but adds that " [t]hese additional funds need to be progressively re-channeled in ways that help build institutional capacity towards a longer-term goal of self-sustaining, universal coverage" [2]. Why universal coverage cannot rely on universal financing is not explained, it is simply assumed. From a report that starts with the contention that " [g]lobalization is putting the social cohesion of many countries under stress", one might have expected a more serious consideration of the option to globalize solidarity in health.

## Competing interests

The author declares that he has no competing interests.
